# Effect of Co Addition on the Microstructure and Mechanical Properties of Sn-11Sb-6Cu Babbitt Alloy

**DOI:** 10.3390/ma17225494

**Published:** 2024-11-11

**Authors:** Zhan Cheng, Meng Wang, Bo Wang, Lei Zhang, Ting Zhu, Ningbo Li, Jifa Zhou, Fei Jia

**Affiliations:** 1 Ningbo Intelligent Machine Tool Research Institute Co., Ltd., China National Machinery Institute Group, Ningbo 315700, China; brazecheng@163.com (Z.C.); zhangl@zrime.com.cn (L.Z.); ningbo721403@163.com (N.L.); 15364914458@163.com (J.Z.); 18804017112@163.com (F.J.); 2 College of Mechanical & Electrical Engineering, Nanjing University of Aeronautics and Astronautics, Nanjing 210016, China; 3 Shenke Slide Bearing Co., Ltd., Shaoxing 311800, China; skjfkfb@163.com

**Keywords:** Babbitt alloy, Co element, microstructure, interface morphology, strength, wear performance

## Abstract

A Babbitt alloy SnSb11Cu6 with 0–2.0 wt.% Co was synthesized using the induction melting process. This study examined the effect of cobalt (Co) on the microstructure, tensile properties, compressive properties, Brinell hardness, and wear properties of SnSb11Cu6 using optical microscopy (OM), scanning electron microscopy (SEM), energy-dispersive X-ray spectroscopy (EDS), X-ray diffraction (XRD), a universal tensile testing machine, a Brinell hardness tester, and a wear testing machine. The results indicate that the optimal quantity of Co can enhance the microstructure of the Babbitt alloy and promote microstructure uniformity, with presence of Co_3_Sn_2_ in the matrix. With the increase in Co content, the tensile and compressive strength of the Babbitt alloy first increased and then decreased, and the Brinell hardness gradually increased with the increase in Co content. The presence of trace Co has a minimal effect on the dry friction coefficient of the Babbitt alloy. When the Co content exceeds 1.5 wt.%, the friction properties of the Babbitt alloy deteriorate significantly. The optimized Babbitt alloy SnSb11Cu6-1.5Co was subsequently fabricated into wires, followed by conducting cold metal transfer (CMT) surfacing experiments. The Co element can promote the growth of interfacial compounds. The microstructure at the interface of the Babbitt alloy/steel is dense, and there is element diffusion between it. The metallurgical bonding is good, and there are serrated compounds relying on the diffusion layer to extend to the direction of the additive layer with serrated compounds extending and growing from the diffusion layer to the additive layer. Overall, Babbitt alloys such as SnSb11Cu6 exhibit improved comprehensive properties when containing 1.5 wt.% Co.

## 1. Introduction

Babbitt alloys are extensively utilized in critical bearing components and steel bearing linings, including turbine shafts in nuclear power plants, internal combustion engine spindles, intermediate bearings, and connecting rod bearings [1,2]. This is attributed to its excellent embedment, compliance and anti-seizure properties [3,4]. However, as machinery and equipment continue to exhibit high performance, their usage is progressively extending to high-speed, heavy-load, high-pressure, and other applications [5,6,7]. This trend unavoidably leads to a reduction in their service life, attributed to their limited load-carrying capacity and inadequate heat resistance [8,9]. Therefore, the development of high-performance Babbitt alloy materials is crucial.

Currently, cold metal transition (CMT) welding holds significant advantages in the production of high-performance sliding bearings [10]. It fulfills the demand for high-performance materials in modern industry. However, the processing difficulty of Babbitt wire is limited by the influence of alloy composition and wire diameter. Among them, the processing difficulty of Babbitt alloy wire with a thin wire diameter containing high antimony and copper components is greatly increased [11]. This seriously limits the development of Babbitt additive manufacturing technology in the field of sliding bearings.

To improve the performance of the Babbitt alloy, researchers have carried out a lot of research on the composition and preparation process of tin-based Babbitt alloys. Zhao [12] conducted a study on the arc deposition process of a Sn Babbitt alloy with 0–2 wt.% of zinc (Zn) on the surface of a steel substrate. The researchers observed that the addition of Zn resulted in a significantly uniform distribution of hard phase SnSb and Cu_6_Sn_5_, leading to improved hardness, strength, and bonding properties. Nikolay [13] proposed the utilization of multiwalled carbon nanotubes as a modifier to suppress the growth of reinforcing particles and enhance the fatigue resistance of bainite coatings. This was achieved by preparing composite particles of bainite powder and multiwalled carbon nanotubes through planetary milling. Subsequently, these particles were deposited onto the substrate surface using a plasma melting process, leading to an enhancement in frictional properties. Ramadan [14] used stir casting to create a bimetallic bearing material by adding nano alumina to a Babbitt alloy and casting it onto carbon steel. The study found that alumina nanoparticles changed Cu_6_Sn_5_ shape, enhancing bond strength and wear resistance. It was observed that a portion of silver (Ag) in Sn-based solid solution underwent solidification within the solution, while another portion combined with the Sn atoms to generate Ag_3_Sn phase, thereby contributing to precipitation strengthening [15].

The aforementioned study employs a range of methods to achieve doping modification of a Babbitt alloy through the addition of metal elements or micro/nano components. Previous research has demonstrated that the incorporation of cobalt (Co) into Sn alloy can enhance the stability of the microstructure and the mechanical properties of the alloy [16,17,18]. However, there is no existing report on the effect of trace Co on the characteristics of Sn-based Babbitt alloy.

Based on the above research, SnSb11Cu6 alloys with different Co contents were prepared by induction melting. Then, the modified Babbitt alloy welding wire was prepared. The effects of Co addition on the microstructure, interface morphology, tensile strength, compressive strength, Brinell hardness, and friction and wear properties of SnSb11Cu6 alloys were systematically studied in order to provide a reference for obtaining a Babbitt alloy with excellent performance and promote the popularization and application of CMT welding technology in the preparation of Babbitt alloy bearings.

At present, the practical application of making a Babbitt alloy into welding wire in CMT (cold metal transfer) additive manufacturing technology is mainly reflected in its use as a filling material for manufacturing or repairing worn or damaged bearing components. Currently, ZSnSb8Cu4 is the main Babbitt alloy welding wire successfully marketed for CMT additive manufacturing. ZSnSb11Cu6 and ZSnSb12Cu6Cd1, which improve performance with doping elements such as Ni, Cd, and As, are gradually being introduced into the market.

## 2. Materials and Methods

This experiment utilized Sn ingots, antimony (Sb) ingots, copper (Cu) particles, and Co blocks, all of which had a purity of 99.9%. Babbitt alloys, SnSb11Cu6 with 0–2.0 wt.% Co, were prepared by adjusting the content of cobalt element. The chemical composition is presented in Table 1.

**Figure 1:**
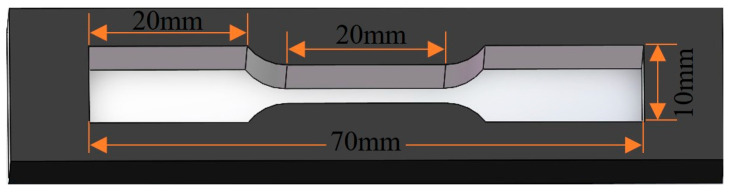
Casting mold of tensile strength sample.

The smelting process involves induction heating and nitrogen protection at a temperature of 550 °C for a duration of 60 min. Throughout the process of melting, dried charcoal was utilized as the covering agent. Following the heat preservation process, the temperature was lowered to 480 °C, at which point ammonium chloride and zinc chloride were introduced to purify the molten substance. The high borosilicate glass rod was stirred for 5 min, and the surface scum was removed by using a slag scoop. Subsequently, the ingot was poured into the preheated tensile sample and the extrusion sample mold, and the ingot structure was obtained by air cooling to room temperature. The tensile sample mold is shown in Figure 1.

The interface was cut between the ingot and the additive using wire cutting. Following coarse grinding, fine grinding, and subsequent fine polishing, the specimen was subjected to discoloration through corrosion using a 4% nitric acid alcohol solution. A Zeiss optical microscope and Zeiss Evo10 scanning electron microscope equipped with Oxford Ultim 40 energy-dispersive X-ray spectroscopy (EDS) (ZEISS Group, Oberkochen, Germany) were employed for macro and micro structure observation, and analysis of the micro components. The intercepted ingot casting machine underwent processing to create standard tensile samples and cylindrical compression samples. The tensile and compressive strength of the specimens were tested by a universal testing machine (MTS Systems Corporation, Eden Prairie, MN, USA) according to the national standards GB/T 228.1-2010 [19] and GB/T 16748-1997 [20]. The displacement rate of the beam during the experiment was 2 mm/min. The Brinell hardness of the material was assessed in accordance with the national standard GB/T 231.1-2009 [21] for metallic materials. The Brinell hardness test (Laizhou Huayin Test Instrument Co., Ltd., Laizhou, China) was conducted as follows: Part 1: Test methods. The loading time was 60 s and the load was 2452 N. Each sample underwent hardness testing five times, and the average values were calculated. The coefficient of friction was determined using HT-1000 High Temperature Friction and Wear Test (Lanzhou Zhongke Kaihua Technology Development Co., Ltd., Lanzhou, China). The experiment utilized 45 steel balls as the grinding material, with a load of 150 g, a loading time of 20 min, a motor frequency of 100 rpm, and a friction radius of 2 mm.

The ingot underwent a series of processes including cutting, peeling, extrusion, stretching, and reduction to produce an alloy wire with a diameter of 1.60 mm. The arc deposition process test was carried out on a Q235 steel plate with a size of 300 mm × 300 mm × 30 mm by using a CMT welding machine (Austrian Fronius company, Wels, Austria) produced by the Austrian Fronius company and Swiss ABB manipulator. Prior to the experiment, the steel plate underwent pickling to eliminate oil and rust from its surface, and the arc deposition process parameters are detailed in Table 2.

## 3. Experimental Results and Analysis

### 3.1. Microstructure Analysis

**Figure 2:**
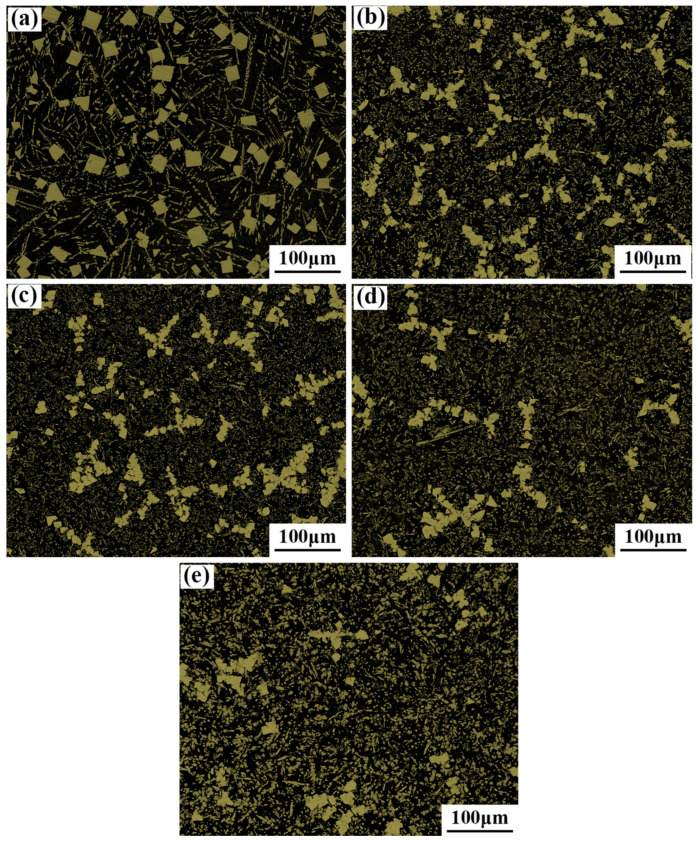
OM microstructure of Babbitt alloy SnSb11Cu6 with different Co contents: (**a**) 0 wt.% Co; (**b**) 0.5 wt.% Co; (**c**) 1.0 wt.% Co; (**d**) 1.5 wt.% Co; (**e**) 2.0 wt.% Co.

**Figure 3:**
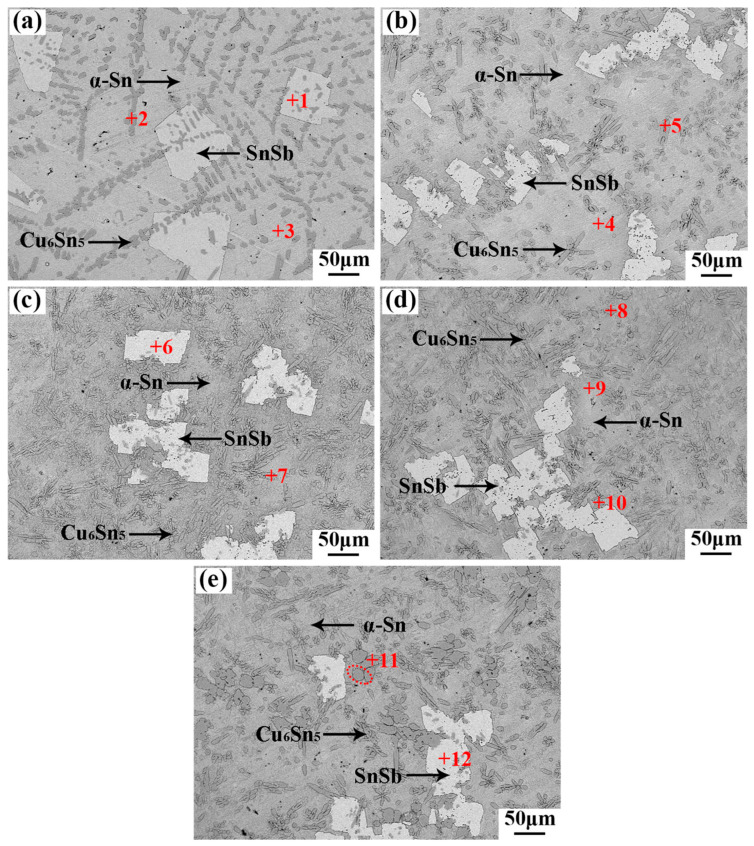
BSE microstructure of Babbitt alloy SnSb11Cu6 with different Co contents: (**a**) 0 wt.% Co; (**b**) 0.5 wt.% Co; (**c**) 1.0 wt.% Co; (**d**) 1.5 wt.% Co; (**e**) 2.0 wt.% Co.

**Figure 4:**
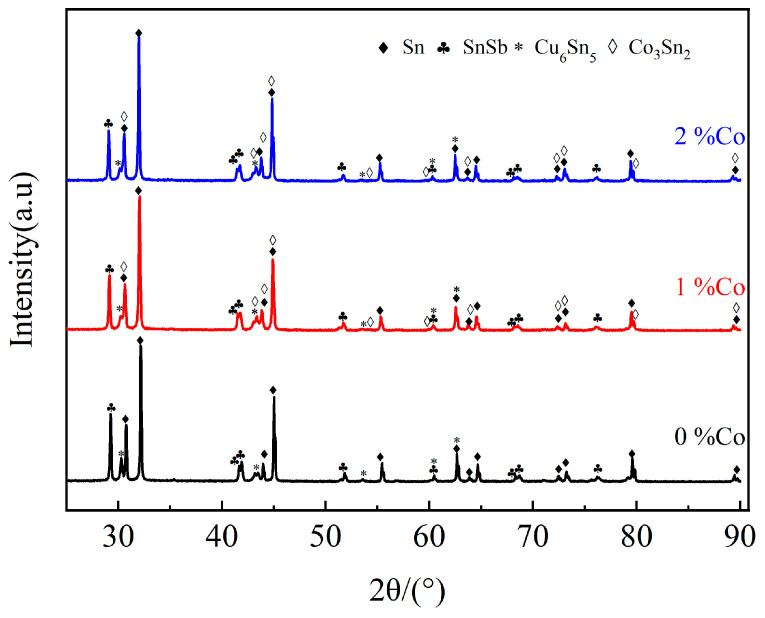
XRD pattern of Babbitt alloy SnSb11Cu6 with different Co contents (0 wt.%, 1.0 wt.%, 2.0 wt.%).

The properties of the alloy were determined by its microstructure. The microstructure and phase composition of the Babbitt Babbittalloy SnSb11Cu6 with 0–2.0 wt.% Co were analyzed. The optical microscopy (OM), scanning electron microscopy (SEM), and X-ray diffraction (XRD) analysis results are depicted in Figure 2, Figure 3 and Figure 4. The microstructure of the Babbitt alloy, as depicted in Figure 2a, predominantly consists of the α-Sn solid solution matrix, SnSb intermetallic compound phase composed of Sn and Sb, and dispersed needle-like and star-like Cu_6_Sn_5_ within the soft matrix. The soft phase α-Sn solid solution, known as the concave, exhibits favorable potential, compliance, and resistance to blockage. On the other hand, the hard phases SnSb and Cu_6_Sn_5_ act as convex elements, creating a small gap between the shaft and the bearing bush. This configuration facilitates the transportation of lubricating medium and contributes to friction reduction.

During the early solidification nucleation process, the Cu_6_Sn_5_ phase first solidified and precipitated. Subsequently, as the crystallization core of the SnSb phase, it exists in the liquid phase melt in the form of a skeleton to prevent the lighter square SnSb phase from floating up. As depicted in Figure 2b–e, the addition of 0.5–2 wt.%. Co passivates the sharp phase angle of SnSb in the microstructure, causing a gradual transformation from square blocks to ellipsoids. Additionally, there is a tendency for these ellipsoids to connect with each other, forming structures resembling flowers. Concurrently, there is a gradual decrease in the content of the SnSb phase. Furthermore, the needle-shaped Cu_6_Sn_5_ undergoes a transformation into small worm-like particles, which are then evenly distributed within the α-Sn solid solution matrix. When the carbon content exceeds 1.5 wt.%. The size of the fine worm-like Cu_6_Sn_5_ particles in the structure begins to coarsen, and the SnSb phase also changes from the trend of mutual aggregation and connection into a star to a dispersed distribution in the matrix, as depicted in Figure 2e.

**Figure 5:**
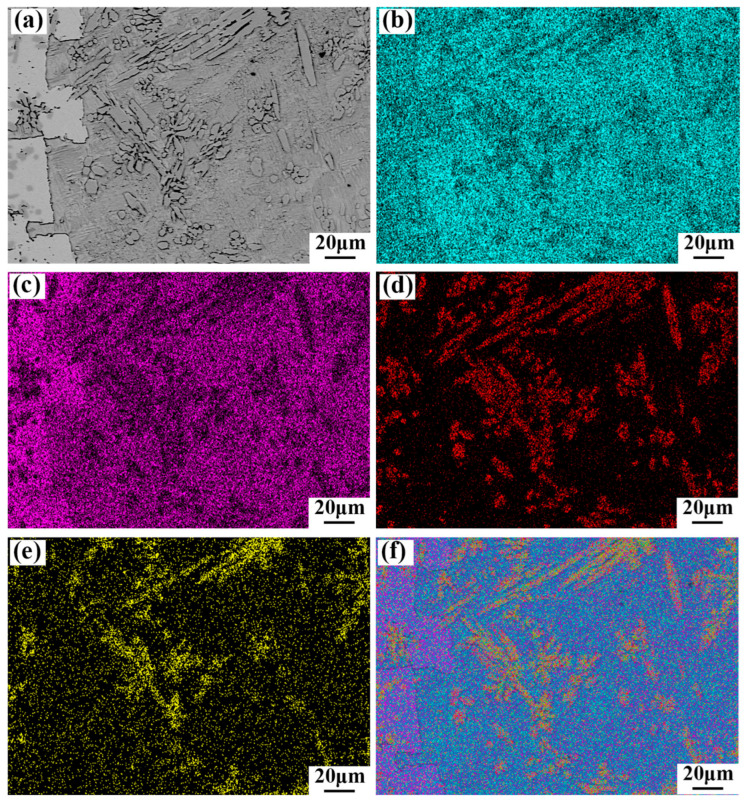
Elemental distribution in microstructure of SnSb11Cu6-1.5Co alloy using BEI and EDS: (**a**) BEI; (**b**) Sn map; (**c**) Sb map (**d**); Cu map; (**e**) Co map; and (**f**) combined elemental composition map.

The structure of the electron microscope was subjected to further analysis, as depicted in Figure 3. The combination of the EDS results in Table 3 and the backscattered electron imaging (BEI) results depicted in Figure 5 for the SnSb11Cu6-1.5Co alloy indicates a uniform distribution of the four elements within the alloy, with no discernible agglomeration. Furthermore, the distribution of Co in the microstructure aligns with that of Cu. Additionally, a portion of Sb undergoes a reaction with Sn to create a brittle SnSb phase, while the remaining portion dissolves into the α-Sn based solid solution to enhance the matrix.

When the added amount of the Co element reaches 2.0 wt.%, a gray-black ellipsoidal compound appears in the tissue, as shown in Figure 2 at point 11. Based on its elemental composition and the Cu-Sn phase diagram, as well as the XRD pattern depicted in Figure 4, the compound is identified as Co_3_Sn_2_. Owing to its high melting point of 1495 °C, Co is expected to be the primary element to precipitate as the crystallization core during the solidification process. At the identical solidification temperature, the non-spontaneous nucleation substrate on which the post-precipitated atoms can attach is relatively increased. This reduces the diffusion distance and nucleation resistance of the post-precipitated atoms, which is conducive to a large number of nucleation in the dendrite region. Subsequently, the Cu_6_Sn_5_ phase crystallizes from the melt in the form of a network. When the temperature is further reduced, the square-shaped β phase (SnSb) begins to precipitate, and the network-like pre-precipitated phase can inhibit the floating of the lighter β phase (SnSb). Then, the tin-based α solid solution begins to solidify. Upon solidification, the alloy’s microstructure becomes finer and more uniform, resulting in a significant refinement of the size of the SnSb phase.

### 3.2. Analysis of Tensile and Compressive Strength

**Figure 6:**
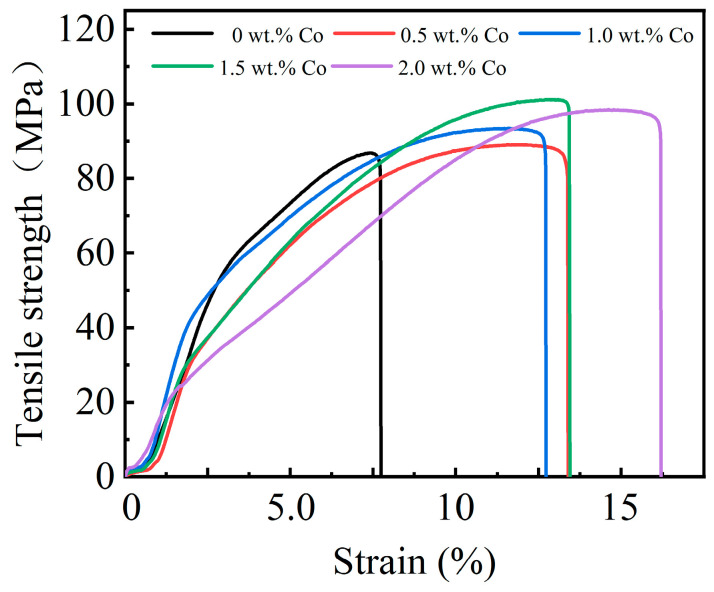
Co content effect on tensile strength of Babbitt alloy SnSb11Cu6 with 0–2.0 wt.% Co.

**Figure 7:**
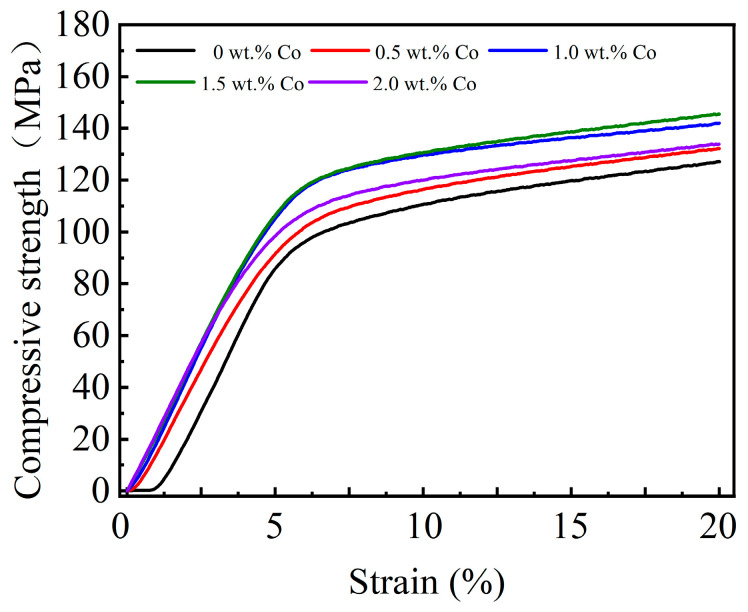
Co content effect on compressive strength of Babbitt alloy SnSb11Cu6 with 0–2.0 wt.% Co.

As a bearing liner, the Babbitt alloy must possess sufficient strength and hardness to endure the substantial pressure exerted by the shaft diameter. The tensile strength and compressive strength of the Babbitt alloy SnSb11Cu6 with 0–2.0 wt.% Co were evaluated using a universal tensile testing machine, and the results are presented in Figure 6 and Figure 7.

The addition of trace Co has been observed to result in a significant increase in the tensile strength and compressive strength of the Babbitt alloy. When the Co content is approximately 1.5 wt.%, the tensile and compressive strengths peak at 101.2 MPa and 141.9 MPa, respectively. These values are 16.8% and 11.6% higher than those of the Babbitt alloy SnSb11Cu6 (86.8 MPa and 127.1 MPa). As the Co content increases to 2.0 wt.%, there is a decrease in both the tensile strength and compressive strength. The observed phenomenon can be attributed to the incorporation of an optimal quantity of Co. On the one hand, part of the Sn-based solid solution has a solid solution strengthening effect. Meanwhile, the remaining portion of Co regulates the Cu_6_Sn_5_ compound from a continuous star to a point-like uniform distribution in the entire matrix. This, in turn, enhances the uniformity of Cu_6_Sn_5_ distribution within the structure. When subjected to external load, the strengthened solid solution and refined grains will increase the resistance of dislocation movement, making it difficult to advance slip. Therefore, the strength of the alloy is increased. After adding too much Co element, the size of the hard brittle phase Cu_6_Sn_5_ compound in the structure increases significantly. At the same time, multilateral Co_3_Sn_2_ compounds also appeared in the structure. This leads to an increase in the overall hardness and brittleness of the structure.

When subjected to an external load, the less coarse grains in the unit volume, the less the plastic deformation generated by the external load can be dispersed to each grain. As a result, it is difficult to uniformly transfer the internal deformation of the metal, resulting in local stress concentration. Then, the strength decreases and breaks first. Therefore, it is essential to manage the quantity of Co in order to modulate the dimensions of Cu_6_Sn_5_ and the concentration of Co_3_Sn_2_ in the structures.

### 3.3. Analysis of Brinell Hardness

**Figure 8:**
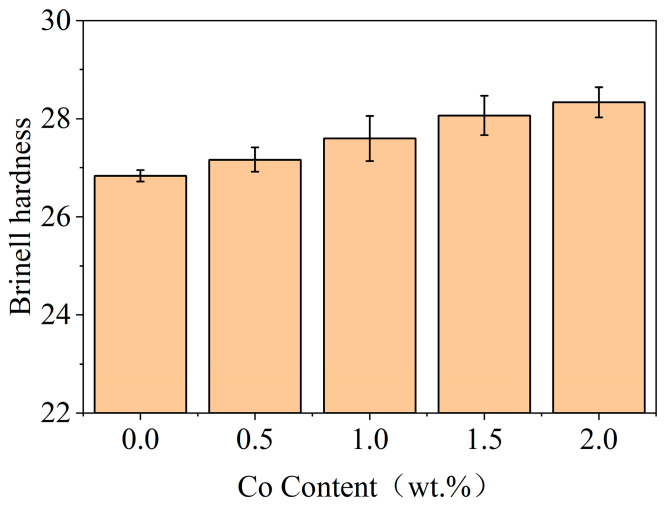
Co content effect on Brinell hardness of Babbitt alloy SnSb11Cu6 with 0–2.0 wt.% Co.

The hardness of the Babbitt layer plays a crucial role in its resistance to deformation during operation and has a direct impact on the friction and wear characteristics of the workpiece. A Brinell hardness tester was utilized for assessing the hardness of the Babbitt alloy, and the corresponding results are presented in Figure 8. The Brinell hardness of SnSb11Cu6 alloy was determined to be 26.8 in the absence of Co. The hardness exhibited a gradual increase with the increase in Co content. At a Co content of 2.0 wt.%, the hardness reached 28.3, representing a 5.6% increase compared to that of SnSb11Cu6. The increase in hardness is attributed to the synergistic effect of solid solution strengthening and dispersion strengthening, as indicated by the analysis of the microstructure in Figure 2.

After the addition of the Co element, the SnSb phases in the structure have a tendency to connect with each other to form a flower shape. With the rise in Co content, the hard brittle phase Cu_6_Sn_5_ compound in the structure changes from a slender strip to an elliptical worm-like shape, the size continues to increase, and the distribution gradually becomes uniform. Furthermore, the emergence of the new Co_3_Sn_2_ phase within the microstructure is expected to contribute to the enhanced hardness of the alloy. Generally, the proportion of the hard phase in the alloy gradually increases following the addition of the Co element. When subjected to an external load, the hard phase of the protrusion increases relatively, so that the hardness gradually increases with the increase in Co content.

### 3.4. Analysis of Wear Properties

**Figure 9:**
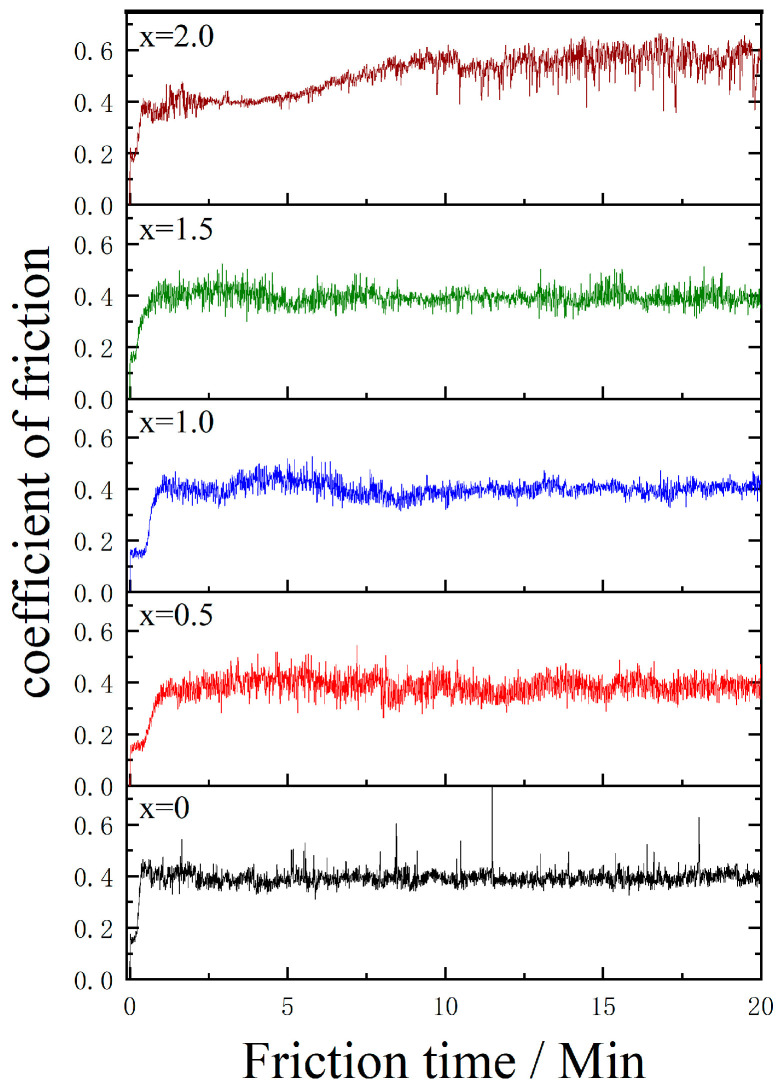
Co content effect on friction coefficient of Babbitt alloy SnSb11Cu6 with 0–2.0 wt.% Co.

The friction coefficient between the modified Babbitt alloy and the stainless steel ball was tested by a friction and wear tester. The results of the friction coefficient for dry friction are depicted in Figure 9. It has been observed that under dry friction conditions, the four friction coefficient curves show minimal variation when the Co content ranges from 0 wt.% to 1.5 wt.%. The coefficients range between 0.37 and 0.40. When the Co content reaches 2.0 wt.%, the friction system stabilizes at approximately 0.4 within 0 min to 7 min. The friction coefficient rises to approximately 0.56 and then reaches a stable state as the duration of friction increases.

The wear resistance of tin-based Babbit alloys is closely related to the thickness and distribution of hard phases in the microstructure [22]. During the wear process, the SnSb phase and Cu_6_Sn_5_ phase, as hard particles, bulge upward to play a supporting role, while the soft matrix α-Sn phase sags downward to play a latent role. When the SnSb phase and Cu_6_Sn_5_ phase are present in small, uniformly distributed quantities within the eutectic matrix, it is possible to achieve outstanding service performance. Combined with the microstructure in Figure 2, it can be seen that the size of the square SnSb phase in the microstructure decreases after the addition of 0–1.5 wt.% Co element, and the hard and brittle Cu_6_Sn_5_ phase is fine and evenly distributed in the whole matrix. When the content of the Co element reaches 2.0 wt.%, the size of the hard Cu_6_Sn_5_ phase in the microstructure increases significantly and is densely distributed. This leads to a decrease in the content of the soft phase in the matrix and reduces the plasticity of the material. Therefore, the embedment and compliance of the material are reduced, which easily causes shedding of the hard phase in the Babbitt alloy and worsens the wear. According to Archard’s law, there is a direct proportionality between the wear resistance of a material and its hardness. The greater the hardness of a material, the greater its resistance to deformation, and consequently, its wear resistance is also increased [23]. During the friction process, the embedding performance of the brittle hard particle phase is reduced, which increases the risk of falling off from the matrix and forming abrasive wear. Therefore, it is necessary to control the size and distribution of the hard phase in the tissue.

### 3.5. Analysis of Interface Morphology

**Figure 10:**
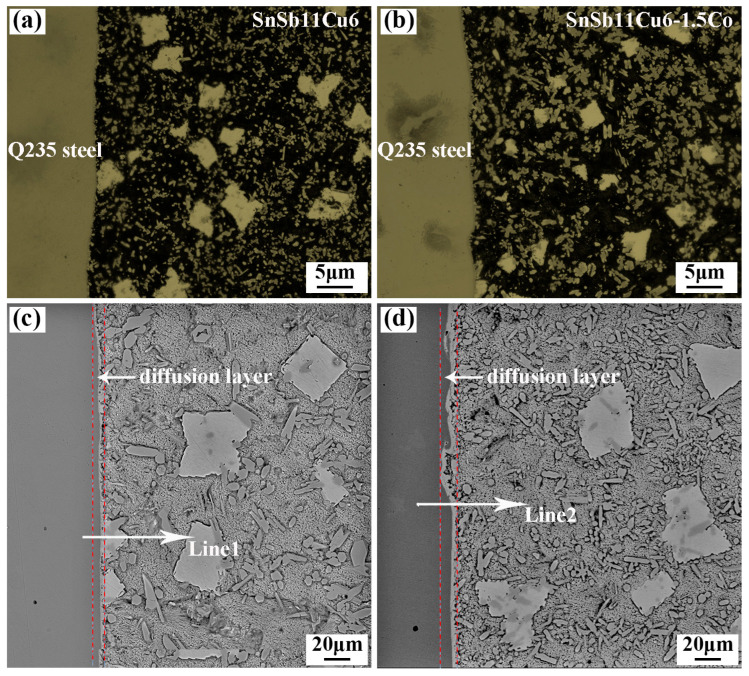
Microstructural interface of Q235 steel/SnSb11Cu6 and Q235 steel/SnSb11Cu6-1.5Co Babbitt alloys: (**a**,**b**) OM; (**c**,**d**) SEM.

**Figure 11:**
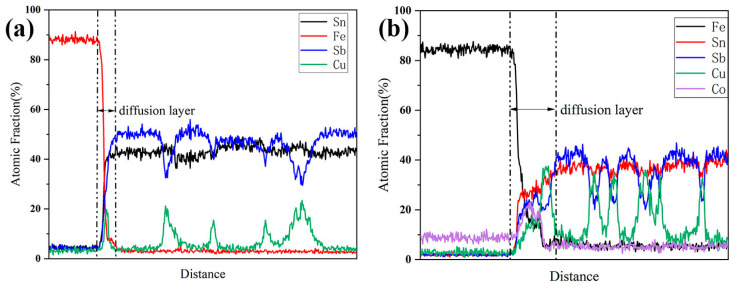
Elemental distribution analysis of Q235 steel/SnSb11Cu6 and Q235 steel/SnSb11Cu6-1.5Co Babbitt alloys: (**a**) SnSb11Cu6; (**b**) SnSb11Cu6-1.5Co.

The Babbitt alloy containing 1.5 wt.% of Co was fabricated into alloy wires with a diameter of 1.6 mm. Subsequently, a CMT welding machine was utilized to perform overlay welding on the steel plate, and the morphology and element distribution of the Babbitt alloy/steel interface are shown in Figure 10 and Figure 11.

It can be seen from Figure 10a,b that the two Babbitt/steel interfaces are continuous and well formed. There are no macroscopic defects such as pores, slag inclusions, and cracks. From the electron microscope morphology of the Babbitt alloy/steel interface in Figure 10c, it can be seen that a relatively flat metallurgical bonding line is formed between the Babbitt alloy and the steel matrix, and there is a serrated compound relying on the interface growth at the Babbitt alloy/steel interface. Further EDS line scanning was performed on the interface, and it was found that the element content from the steel side interface to the additive layer showed a certain concentration gradient. The content of iron decreased gradually, and the content of Sn, Sb and Cu increased gradually, as shown in Figure 11a. Following the addition of 1.5 wt.% of Co, the thickness of the compound layer at the Babbitt alloy/steel interface increased from 0.95 μm to 2.14 μm. The size of the serrated compound is notably larger, as depicted in Figure 10d. When subjected to external load, these serrated compounds can play a pinning role by inserting into the Babbitt alloy matrix relying on the interface layer to improve the holding force at the interface [24]. The energy spectrum analysis of the interface is carried out, and the results are shown in Figure 11b. It was found that from the steel side interface to the additive layer, the iron element gradually decreased, the content of tin, antimony and copper gradually increased, and the Co element had a convex peak at the interface. This indicates that the Co element is enriched at the interface, indicating that the addition of Co element can promote the growth of interfacial compounds.

## 4. Discussion

In this study, the microstructure, tensile and compressive strength, hardness, and wear properties of the Babbitt alloy SnSb11Cu6 with different contents of cobalt (Co) were comprehensively analyzed. Then, the experimental results were further compared with the findings of other researchers to verify the trends and phenomena in this study.

In terms of the microstructure, with the increase in Co content, the shape of the SnSb phase gradually changes from square blocks to ellipses. And there is a trend of connecting to form a flower-like structure. This observation is consistent with that of Wang [25] and Liu [16] that adding trace amounts of Co to Sn-0.7Cu alloy can alter the morphology of the phase, thus similarly, affecting the mechanical properties of the alloy. In addition, we found that when the Co content exceeds 1.5 wt.%, the size of the Cu_6_Sn_5_ phase begins to coarsen. An analysis of the interface morphology revealed that the Babbitt alloy with 1.5 wt.% Co added formed a thicker compound layer at the interface with the steel substrate, which is consistent with the research results of Tang [26]. They pointed out that the addition of Co can promote the growth of interfacial compounds, thereby improving the binding strength of the interface.

The mechanical properties of the Co-modified Babbitt alloy were studied. When the Co content was about 1.5 wt.%, the tensile and compressive strength of the Babbitt alloy reached the peak. This finding is consistent with the research results of Zhang [27,28,29], who found that the strength of the Babbitt alloy can be significantly improved by adding an appropriate amount of Co. When the Co content increased to 2.0 wt.%, the strength decreased. This may be due to the increase in the size of the hard and brittle Cu_6_Sn_5_ phase and the formation of a new Co_3_Sn_2_ phase. Excessive Co will lead to an increase in brittleness of the alloy. In addition, with an increase in Co content, the hardness of the Babbitt alloy gradually increases, which is consistent with the research of Fan [29], who found that the addition of Co can improve the hardness of the alloy through the synergistic effect of solution strengthening and dispersion strengthening. Our results further confirm this. In particular, when the Co content is 2.0 wt.%, the increase in hardness is most significant.

By analyzing the wear properties, we found that the friction coefficient increased significantly when the Co content was 2.0 wt.%. This is because excessive Co will lead to coarsening of the Cu_6_Sn_5_ phase, which will affect the wear resistance of the alloy. This result is consistent with the research of Zhu and Hao [30,31]. They point out that an increase in the hard phase size will lead to an increase in the friction coefficient. Our study further shows that the size and distribution of the hard phase have an important effect on the wear properties of the Babbitt alloy. This is consistent with Archard’s law [23]: the hardness of the material is proportional to its wear resistance.

In summary, our research findings are consistent with those of other researchers, further verifying the effect of Co addition on the microstructure and properties of Babbitt alloys. This study provides a valuable reference for the optimization of Babbitt alloys.

## 5. Conclusions

(1) The Co element can significantly refine the microstructure of the Babbitt alloy, improve the uniformity of the structure, and exist in the matrix as Co_3_Sn_2_ compound. As the concentration of Co content increases, the sharp angle passivation of β-SnSb phase transforms into ellipsoidal connection, and the content of SnSb phase decreases gradually. The needle-like Cu_6_Sn_5_ phase is transformed into small worm-like particles, which are dispersed in the matrix α solid solution. When the Co content exceeds 1.5 wt.%, the Cu_6_Sn_5_ phase is significantly coarsened. The presence of Co can enhance the formation of interfacial compounds. The interface exhibits a compact structure and strong metallurgical bonding. The serrated compound at the interface depends on the diffusion layer to elongate and expand towards the Babbitt alloy layer.
(2) The tensile strength and compressive strength of Babbitt alloy exhibit a pattern of initial increase followed by decrease as Co content increases. The maximum values of 101.2 MPa and 141.9 MPa were attained at a Co content of 1.5 wt.%, representing an increase of 16.8% and 11.6%, respectively, compared to Babbitt alloy SnSb11Cu6 (86.8 MPa). The Brinell hardness gradually increases with the increase in Co element content. At a Co content of 2.0 wt.%, the alloy achieved its peak value (28.3), representing a 5.6% increase compared to Babbitt alloy SnSb11Cu6.
(3) The trace addition of Co element has minimal impact on the dry friction coefficient of Babbitt alloy. When Co content exceeds 1.5 wt.%, there is a significant deterioration in friction performance.
(4) In general, the addition of 1.5 wt.% of Co results in Babbitt alloy SnSb11Cu6 exhibiting favorable comprehensive properties.

## Figures and Tables

**Table 1:** Chemical compositions of Babbitt alloy SnSb11Cu6 with 0–2.0 wt.% Co.

Alloy	Sn	Sb	Cu	Co
1	Bal.	11.11	5.96	0
2	Bal.	11.08	5.99	0.49
3	Bal.	11.14	6.07	1.07
4	Bal.	11.09	6.01	1.48
5	Bal.	11.12	5.98	2.03

**Table 2:** Process parameters employed in CMT arc deposition of Sn-based Babbitt alloy.

Stacking Number	Welding Voltage/V	Welding Current/A	Wire Feed Rate/m·min^−1^	Welding Speed/mm·min^−1^
1	18.2~18.4	110~120	6.1~6.6	600
2	11.9~12.0	60~70	6.7~7.6	480

**Table 3:** Elemental composition of EDS at each point in Figure 3 (at.%).

Point	Sn	Sb	Cu	Co	Possible Phase
1	57.5	42.5	-	-	SnSb
2	44.9	3.7	51.5	-	Cu_6_Sn_5_
3	86.1	13.9	-	-	(Sn)
4	87.4	12.6	-	-	(Sn)
5	42.3	5.5	43.6	8.5	Cu_6_Sn_5_
6	56.6	43.4	-	-	SnSb
7	44.0	7.6	35.9	12.4	Cu_6_Sn_5_
8	39.4	8.0	36.8	15.7	Cu_6_Sn_5_
9	81.4	18.6	-	-	(Sn)
10	44.0	4.1	48.7	3.1	Cu_6_Sn_5_
11	35.0	11.3	22.9	30.8	Co_3_Sn_2_, Cu_6_Sn_5_
12	57.8	42.2	-	-	SnSb

## Data Availability

The original contributions presented in the study are included in the article, further inquiries can be directed to the corresponding authors.
